# Psychiatric medication and physical performance parameters – Are there implications for treatment?

**DOI:** 10.3389/fpsyt.2022.985983

**Published:** 2022-09-06

**Authors:** Anna Hirschbeck, Douglas Silva Leao, Elias Wagner, Alkomiet Hasan, Astrid Roeh

**Affiliations:** ^1^Department of Psychiatry, Psychotherapy and Psychosomatics, Bezirkskrankenhaus Augsburg, Medical Faculty, University of Augsburg, Augsburg, Germany; ^2^Department of Psychiatry and Psychotherapy, University Hospital, Ludwig Maximilian University of Munich, Munich, Germany

**Keywords:** psychiatric medication, psychotropic drugs, physical performance, athletic performance, sport, exercise, fitness, systematic review

## Abstract

**Introduction:**

The impact of psychiatric medications and their enhancing or impairing effects on physical performance remains inconclusive. Therefore, with this systematic review we provide a comprehensive overview of frequently used psychotropic drugs and their effects on physical performance for the purpose of providing empirical information and deriving prescription and therapy recommendations for clinical practice.

**Methods:**

We systematically searched PubMed, PsycInfo, and Cochrane databases and extracted human studies investigating the effect of psychotropic drugs on parameters associated with the level of physical performance, such as exercise time, oxygen consumption, heart rate, muscle contraction or blood lactate concentration in physically healthy participants. 36 studies - comprising a broad range of psychotropic agents, such as antidepressants, antipsychotics, sedatives, and stimulants - were selected for final analyses.

**Results:**

Most studies (*N* = 32) were randomized controlled trials (RCT) with a double-blind crossover design. Antidepressants (*N* = 21) were the most frequently studied drug class, with contradictory results e.g., performance enhancement in warm environment but not in temperate conditions for bupropion or inconsistent findings between studies for other antidepressants. Antipsychotics (*N* = 3) mainly showed impairing effects on physical performance, while stimulants (*N* = 4) were often performance-enhancing. Sedatives (*N* = 9) may cause a hangover effect.

**Conclusion:**

The examined studies with heterogeneous design showed different effects of psychiatric medications on physical performance. Antipsychotics seemed to be performance impairing, while the findings for antidepressants and sedatives were more inconsistent. Stimulants were the only group with consistent performance-enhancing effects. However, most studies were conducted with a small sample size (*N* < 10), mostly in well-trained subjects rather than in patients with psychiatric disorders, and most studies used single-dose designs. These issues impede the formulation of generalized conclusions for treatment regimes and should therefore be considered in further longitudinal studies for clinically reliable statements. Nevertheless, answering our research question is quite relevant for clinical practice and therapeutic prescription and should be further investigated especially considering the high drop-out rates in drug treatment.

**Systematic review registration:**

[https://www.crd.york.ac.uk/prospero/display_record.php?RecordID=276103], identifier [CRD42021276103].

## Introduction

Psychopharmacological drugs play a pivotal role in the treatment of severe mental illness. Despite indisputable benefits in treatment regimes, the intake of the medication also bears risks and side-effects. Possible side-effects in terms of exercise performance impairments may include, among others, fatigue, muscle stiffness or weakness and these side-effects can negatively impact physical performance (1, 2).

In the past years, physical exercise has become increasingly important in therapeutic regimes of various psychiatric diseases, e.g., aerobic exercise of moderate-vigorous intensity or in combination with resistance training at a frequency of 2–3 times a week, achieving 150 min of moderate-to-vigorous physical activity (3). Physical exercise in this context is also beneficial for the amount of visceral and epicardial adipose tissue and for factors constituting the metabolic syndrome (4). Therefore, side-effects of psychiatric medications with negative impact on physical performance should be strictly avoided.

Previous scientific background for the effects of psychiatric medication on athletic performance was mainly provided in the population of competitive athletes and healthy people. For example one experimental study with *N* = 6 subjects showed that 70 mg of fluoxetine (selective serotonin reuptake inhibitor = SSRI) reduced the ability to perform prolonged exercise on a bicycle ergometer (5). However, the intake of bupropion (norepinephrine and dopamine reuptake inhibitor = NDRI) increased physical performance in the heat after acute administration (6). Another study examined the intake of tricyclic antidepressants (desipramine 3 mg/kg body weight) in 9 children and 13 adults with no connection to competitive sports. A comparison of treadmill exercise tests before and after a single dose showed no differences in performance, but slight changes in blood pressure and heart rate (7). In summary, the studies were conducted in small sample sizes and results for antidepressive agents were inconclusive. Concerning medication with impact on sleep and anxiety, existing data is also inconsistent. Studies on the so-called Z-substances could not identify impaired athletic performance measured with a 50-m-sprint test after taking two doses of zolpidem 10 mg with *N* = 8 subjects (8), as well as no increased endurance time after a running time test with two doses of zopiclone 7,5 mg in *N* = 8 athletes (9). This is in contrast to buspirone with increased exhaustion after a 45 mg single dose in *N* = 13 athletes after a cycled ergometer test at 80% of maximum oxygen consumption (VO_2_max) (10). Other studies found negative effects of melatonin 6 mg in *N* = 23 subjects on psychomotor performance (11).

The described studies mostly referred to healthy adults. Investigations in children/adolescents and elderly people are rare. One study with *N* = 45 showed that the physical activity of adolescents treated with psychotropic medications was significantly impaired compared to adolescents without medication and to healthy controls (12). Similar results were described among elderly people. A 4-year prospective cohort study conducted at the end of the 1980s with *N* = 885 older women (over 70 years) showed that the regular use of benzodiazepines had a negative effect on physical performance, such as walking speed or balance (13).

The occurrence of distressing side-effects often lead to the discontinuation of psychotropic medication and should be considered during treatment (14). The impairments of physical performance displayed above are not restricted to athletes, but might be relevant in a larger context to all treated patients. For patients, it might be difficult to differentiate between subjective side-effects, such as tiredness or fatigue, and objectively measurable impairments in physical performance. Therefore, there is a need to systematically investigate the impact of psychotropic mediation on physical performance as which might have been neglected in the scientific literature despite its relevance.

With this systematic review we want to provide the first comprehensive overview of frequently used psychotropic drugs (antidepressants, antipsychotics, sedatives, and stimulants) and their effects on physical performance in order to provide founded information and derive prescription and therapy recommendations for clinical practice.

### Objective

To the best of our knowledge, this is the first systematic review summarizing psychopharmacological effects on physical performance parameters. This overview will help to adequately inform patients and clinicians to avoid wrong conclusions regarding the negative impact of psychotropic agents on physical performance.

## Methods

### Search strategy

This systematic review was registered on PROSPERO (CRD42021276103). We systematically searched PubMed, PsycInfo and Cochrane databases with all combination of the following search terms: psychotropic drugs OR psychiatric medication OR serotonin reuptake inhibitor OR antidepressant drugs OR antipsychotic drugs OR dopamine reuptake inhibitor OR antidepressants OR antipsychotics OR sedatives OR anxiolytics OR hypnotics OR mood stabilizer AND athletic performance OR exercise performance OR physical performance OR physical fitness OR exercise testing OR aerobic capacity OR elite athletes.

The database search was last updated on August 05, 2022. All citations were screened for relevance by title in a first step, by abstract in a second step and by full-text in a last step. In all included records, the citations were screened manually for further relevant studies that may have not been detected by the systematic search. The systematic literature search and selection was performed by AH, the selection was afterward reviewed independently by DS. Both AH and DS retrieved the relevant information, and the results were compared. In case of disagreement, a third author (AR) was consulted. The Preferred Reporting Items for Systematic Reviews and Meta-Analyses (PRISMA) were considered.

### Eligibility criteria

We included studies that contain an evaluation of psychotropic drugs on physical performance, such as exercise time, oxygen consumption, heart rate, muscle contraction or blood lactate concentration in physically healthy adult human participants. No limitations were defined for the type of psychiatric disorder and the type of studies. All studies that were published until December 31, 2021, in English language were considered. We excluded studies without specific data about physical fitness, animal, preclinical and molecular studies as well as expert opinions and position statements.

### Quality assessment

Each publication was reviewed using the Scottish Intercollegiate Guidelines Network (SIGN) methodology checklist which assess the internal validity as well as the external validity of our included studies (15). The more the required criteria (e.g., appropriate and clearly focused question, randomization, adequate concealment method, blinding, or percentage of dropout) can be agreed to, the higher the quality is rated. A distinction is made between high quality, acceptable, low quality and unacceptable. We further used the simple Jadad Scale quickly assessing the methodological quality of a clinical trial that included three items (randomization, blinding, and withdrawals/dropouts) and is evaluated with a point system via yes-and-no questions (16).

## Results

**FIGURE 1:**
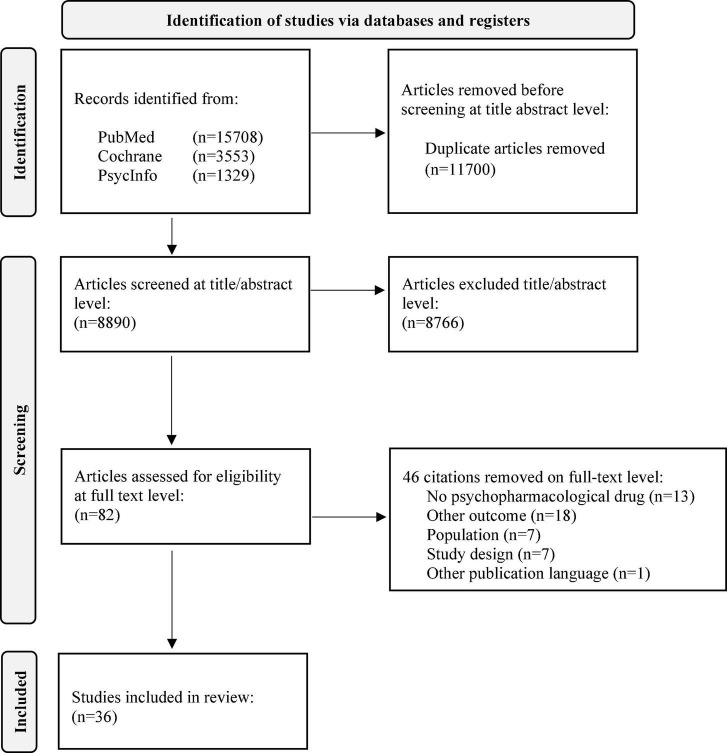
PRISMA flow diagram of included studies.

We were able to include 36 studies in the final analysis (see Figure 1). A detailed description of included publications and study types as well as the reviewed quality scores are shown in Table 1.

**TABLE 1:** List of evaluated psychiatric drugs.

Psychiatric drug		References	Study design	*N*	SIGN	Jadad Scale
Antidepressants	Paroxetine	Kavanagh et al. (21)	Randomized, double-blind, placebo controlled, and crossover design	15	+	3
		Strachan et al. (22)	Randomized, double-blind, placebo controlled, and crossover design	8	++	4
		Strüder et al. (23)	Randomized, double-blind, placebo controlled, and crossover design	10	++	3
		Teixeira-Coelho et al. (24)	Randomized, double-blind, placebo controlled, and crossover design	16	++	4
		Thorstensen et al. (25)	Randomized, double-blind, placebo controlled, and crossover design	15	+	3
		Wilson et al. (26)	Randomized, double-blind, placebo controlled, and crossover design	7	++	5
	Fluoxetine	Meeusen et al. (27)	Randomized, double-blind, placebo controlled, and crossover design	8	++	4
		Parise et al. (19)	Randomized, double-blind, placebo controlled, and crossover design	11+12	++	5
	Sertraline	Bilici et al. (28)	Case control study	38	+	/
	Fluvoxamine					
	Citalopram					
	Bupropion	Cordery et al. (29)	Randomized, double-blind, placebo controlled, and crossover design	9	++	4
		Onus et al. (30)	Randomized single-blind design	8	+	3
		Piacentini et al. (31)	Randomized, double-blind, placebo controlled, and crossover design	8	++	4
		Roelands et al. (20)	Randomized, double-blind, placebo controlled, and crossover design	8	++	4
		Roelands et al. (32)	Randomized, double-blind, placebo controlled, and crossover design	10	++	4
		Watson et al. (6)	Randomized, double-blind, placebo controlled, and crossover design	9	++	4
	Reboxetine	Goekint et al. (33)	Randomized, double-blind, placebo controlled, and crossover design	11	++	4
		Klass et al. (34)	Randomized, double-blind, placebo controlled, and crossover design	10	++	4
		Klass et al. (35)	Randomized, double-blind, placebo controlled, and crossover design	9	+	4
		Piacentini et al. (36)	Randomized, double-blind, placebo controlled, and crossover design	7	++	4
		Roelands et al. (37)	Randomized, double-blind, placebo controlled, and crossover design	9	++	4
	Ritanserin	Meeusen et al. (38)	Randomized, double-blind, placebo controlled, and crossover design	7	++	4
Antipsychotics	Oxypertine	Adamson and Finlay (17)	Randomized, double-blind, and crossover design	5	−	3
	Clozapine	Kim et al. (39)	Cross-sectional study	30 + 15	+	/
	Olanzapine	Perez-Cruzado et al. (40)	Cross-sectional study	62	+	/
	Risperidone					
Sedatives	Benzodiazepine	Charles et al. (18)	Randomized, double-blind, placebo controlled, and crossover design	8 + 27	+	4
		Collomp et al. (41)	Randomized, double-blind, placebo controlled, and crossover design	7	++	4
		Collomp et al. (42)	Randomized, double-blind, placebo controlled, and crossover design	7	++	4
		Ergen et al. (43)	Randomized, double-blind, placebo controlled, and crossover design	24	++	4
		Grobler et al. (44)	Randomized, double-blind, placebo controlled, and crossover design	12	++	4
	Z-drugs	Grobler et al. (44)	Randomized, double-blind, placebo controlled, and crossover design	12	++	4
		Suda et al. (45)	Randomized, double-blind, placebo controlled, and crossover design	12	++	3
		Ito et al. (8)	Randomized, double-blind, placebo controlled, and crossover design	8	++	3
		Ito et al. (46)	Randomized, double-blind, placebo controlled, and crossover design	21	++	3
		Tafti et al. (9)	Randomized, double-blind, placebo controlled, and crossover design	8	++	2
Stimulants	Methylphenidate	King et al. (47)	Randomized, double-blind, placebo controlled, and crossover design	15	−	3
		Klass et al. (34)	Randomized, double-blind, placebo controlled, and crossover design	10	++	4
		Roelands et al. (48)	Randomized, double-blind, placebo controlled, and crossover design	8	++	4
	Others	Westover et al. (49)	Cross-sectional study	245	+	/

N, total sample size; SIGN, ++, high quality; +, acceptable, −, low quality; Jadad Scale, 0–5 (with 5 as the maximum; /, not applicable).

### Study selection

The initial search without further restrictions resulted in 20,590 citations. After elimination of duplicates, 8,890 citations were included for further analysis. The screening on title/abstract level eliminated 8,766 citations (124 remaining). After full-text screening, 36 citations were considered for final analysis. Figure 1 presents the PRISMA chart.

### Study characteristics

For a better overview, we divided the included studies into four groups: antidepressants (*N* = 21), antipsychotics (*N* = 3), sedatives (*N* = 9), and stimulants (*N* = 4). The experimental testing consisted of isokinetic measurements of individual muscle (groups) and bicycle cycling at the individual percentage of VO_2*max*_ or maximum wattage (*W*_*max*_) and physical fitness tests (e.g., vertical jumps, standing jumps, and sprints). Across all RCTs, the mean sample size was *N* < 10 with a minimum of 5 (17) and a maximum of 27 (18). The drugs were mostly administered as a single dose and taken the night before or in the morning of the experiment. Continuous administration was only given in two studies (19, 20).

### Results of individual studies

A detailed summary of all publications including study population, experimental testing design, drug, time of drug intake and relevant physical performance outcomes is provided for antidepressants in Table 2, for antipsychotics in Table 3, for sedatives in Table 4, and for stimulants in Table 5. The statistical data are shown in the Supplementary material.

**TABLE 2:** Antidepressants: experimental testing, drug and measured performance parameters.

References	Sample size	Experimental testing	Drug	Time of drug intake	Performance and physical parameters (*primary/secondary*)	Statistical outcome (*data compared to placebo/all trials*)
Kavanagh et al. (21)	Study 1: *N* = 14 Study 2: *N* = 11 Study 3: *N* = 8	Isometric elbow flexion torque, biceps brachii EMG and triceps brachii EMG was recorded from the dominant limb and were measured during a series of maximal contraction tasks. Study 1: 8 maximal elbow flexions, each maintained until torque declined to less than 60% MVC ≥ 3 s (s). Time-to-task failure was calculated from the onset of elbow flexion torque to the time that torque declined to 60% MVC ≥ 3. Study 2: Voluntary activation was examined for the non-fatigued and fatigued biceps muscle via electrical stimulation of intramuscular fibers (motor nerve). Electrically evoked increases in torque were quantified during maximal contractions (superimposed twitch) and for the relaxed muscle (resting twitches). Study 3: F-waves were obtained from the abductor digiti minimi before and after a series of maximal fifth digit abduction contractions.	Paroxetine 20 mg	4 h before exercise testing	**Primary:** MVC. **Secondary:** Alertness. RPE. Max. torque. Voluntary activation. F-waves.	**Study 1:** MVC: ↑ (4%). Max. torque: 11 N ↑, 3 N ↓. Alertness: ↓. RPE: ↑. **Study 2:** Max. torque: ↑. Voluntary activation, unfatigued muscle: ↑. Voluntary activation, fatigued muscle: ↓. **Study 3:** **2-s MVC.** F-waves: ↓. F-waves area: ↓. F-waves persistence: ↓. **60-s MVC** F-waves: ↓. F-waves area: ↓. F-waves persistence: ↓.
Strachan et al. (22)	*N* = 8	Cycling tests at 60% of VO_2_max until exhaustion in a warm (32°) condition.	Paroxetine 20 mg.	5 h before exercise trials	**Primary:** Time to exhaustion. **Secondary:** Heart rate. Blood lactate. RPE.	Time to exhaustion: →. Heart rate: →. Blood lactate: →. RPE: →.
Strüder et al. (23)	*N* = 10	Cycling with workload (256.0 ± 19.5 W) corresponded to a blood lactate level of 2.0 mmol/l in an incrementally graded exercise test until exhaustion.	Paroxetine 20 mg.	Approx. 5 h before exercise trials.	**Secondary:** Time to exhaustion. Heart rate. Blood lactate.	Time to exhaustion: ↓. Heart rate: →. Blood lactate: →.
Teixeira-Coelho et al. (24)	*N* = 16	Cycling at 50 rpm until voluntary termination of the exercise at an intensity corresponding to 60% Wmax (fatigue protocol).	Paroxetine 10 mg/20 mg/40 mg.	One capsule at 12:00 pm on the experimental testing day.	**Primary:** Exercise time. **Secondary:** Heart rate. Blood lactate. RPE.	Exercise time: ↓ 15% in 20 mg. Heart rate: →. Blood lactate: →. RPE: →.
Thorstensen et al. (25)	*N* = 15	Low-intensity isometric measurement of elbow flexor (unfatigued, fatigued for 30 min at 15% of MVC and recovery).	Paroxetine 20 mg.	One capsule 4 h prior to the commencement of experiments.	**Primary:** Peak torque. **Secondary:** Voluntary activation. Perceived fatigue. Biceps silent period.	Peak torque: →. Voluntary activation: →. Perceived fatigue: ↑. Biceps silent period: ↓.
Wilson et al. (26)	*N* = 7	Cycling at 70% of VO_2_max until exhaustion.	Paroxetine 20 mg.	6 h before each test.	**Primary:** Endurance time. **Secondary:** Relative work load (%VO_2_max). Heart rate. Blood lactate peak. RPE.	Endurance time: ↓. %VO_2_max: →. Heart rate: →. Blood lactate peak: ↓ after 15 min. RPE: →.
Bilici et al. (28)	*N* = 38	Isokinetic measurements of quadriceps and hamstring muscles: 6 maximal repetitions of knee extension and flexion at velocities of 60°/s and 180°/s before and after subchronic antidepressant treatment.	3 months drug therapy of Fluoxetine (20 mg), Sertraline (50 mg), Fluvoxamine (100 mg), and Citalopram (20 mg).	Daily dose.	**Primary:** Isokinetic muscle performance (IMP): Peak torque (PT). Total work (TW). Acceleration time (AT).	PT: ↑. TW: ↑. AT: ↓.
Meeusen et al. (27)	*N* = 8	90-min cycling at 65% Wmax.	Fluoxetine-HCI 20 mg.	Two capsules the night before and the morning of the experiment.	**Primary:** Exercise time. **Secondary:** Blood lactate. RPE.	Exercise time: →. Blood lactate: ↑ in resting. RPE: →.
Parise et al. (19)	Acute study: *N* = 11 Chronic study: *N* = 12	Acute study: repeated 30-s maximal cycling tests (2 tests) (Wingate); Cycling to exhaustion at 80% VO_2_max; Isokinetic measurements of right knee extensor. Chronic study: single 30-s maximal cycling test (Wingate); cycling to exhaustion at 90% VO_2_max; Isokinetic measurements of dorsiflexors.	Acute study: fluoxetine 40 mg. Chronic study: fluoxetine 40 mg.	Acute study: 6 h before testing. Chronic study: daily for 2 weeks.	**Acute study:** **Primary:** MVC. **Secondary:** Peak power. Mean power. Fatigue index. Blood lactate. Time to exhaustion. **Chronic study:** **Primary:** MVC. **Secondary:** Peak power. Mean power. Blood lactate. VO_2_max. Ventilation. Heart rate.	**Acute study:** MVC: ↓. Peak power: →. Mean power: →. Fatigue index: →. Blood lactate: →. Time to exhaustion: →. **Chronic study:** MVC: →. Peak power: →. Mean power: →. Blood lactate: →. VO_2_max: →. Ventilation: ↓. Heart rate: →.
Cordery et al. (29)	*N* = 9	60-min cycling at 60% VO_2_ peak followed by a 30-min performance test, in which participants were asked to complete as much work as possible in a warm (30°C) environment.	Bupropion 600 mg (2 × 2 doses 150 mg).	In two doses the night before and the morning of experiment.	**Primary:** Watts. **Secondary:** Total work. Heart rate.	Watts: ↑. Total work done: ↑. Heart rate: →.
Onus et al. (30)	*N* = 8	30-min-intensity cycling at 50% Wmax in either warm (32°C) or moderate (20°C) ambient conditions followed by a self-paced time trial with each section interspersed with a 30 s maximal sprint at 9, 19 and 29 min. Isokinetic measurements of forearm flexors and knee extensors (rectus femoris, vastus medialis, vastus lateralis, biceps femoris).	Bupropion 600 mg (2 × 300 mg).	One dose the night before and the second dose 3 h prior to each testing session.	**Primary:** Total distance. **Secondary:** Mean power output. Peak power output. Mean speed. Max speed. Heart rate. RPE.	**Fixed intensity:** Total distance: →. Mean power output: →. Peak power output: →. Mean speed: →. Max speed sprints: ↓ in 20°C, ↓ in 32°C. Heart rate: →. RPE: →, ↑in 32°C. **Self-paced:** Total distance: →. Mean power output: →. Peak power output: →. Mean speed: →. Max speed: →. Heart rate: →. RPE: ↑ in 32°C.
Piacentini et al. (31)	*N* = 8	Cycling tests (time trial) starting at 65% Wmax until the participants completed a predetermined amount of work (equal to about 90 min cycling at 65% Wmax as fast as possible).	Bupropion 600 mg (2 × 300 mg).	The night before and the morning of the experiment.	**Secondary:** Exercise time. Heart rate. Blood lactate. RPE.	Exercise time: →. Heart rate: →. Blood lactate: →. RPE: →.
Roelands et al. (20)	*N* = 8	60-min cycling at 55% of Wmax followed by a time trial until the participants completed a predetermined amount of work (equal to about 30 min cycling at 75% Wmax as fast as possible) in warm (30°C) condition.	Bupropion 3 × 150 mg; Bupropion 7 × 300 mg (2 × 150 mg).	One pill (150 mg) for each of the first 3 days and two capsules for the remaining 7 days (one pill in the morning, the second in the afternoon).	**Primary:** Exercise time. **Secondary:** Mean power output (W). Heart rate. RPE.	Exercise time: →. Mean power output: →. Heart rate: →. RPE: →.
Roelands et al. (32)	*N* = 10	60-min cycling at 55% of Wmax followed by a time trial until the participants completed a predetermined amount of work (equal to about 30 min cycling at 75% Wmax as fast as possible) in warm (30°C) condition.	Bupropion (2× à 150 mg/225 mg/300 mg).	The night before and the morning of the experiment.	**Primary:** Exercise time. **Secondary:** Heart rate. RPE.	Exercise time: ↓ in 300 mg (faster). Heart rate: ↑ in 300 mg. RPE: →.
Watson et al. (6)	*N* = 9	60-min cycling at 55% of Wmax followed by a time trial until the participants completed a predetermined amount of work (equal to about 30 min cycling at 75% Wmax as fast as possible) in temperate (18°C) or warm (30°C) conditions.	Bupropion 600 mg (2 × 300 mg).	The night before and the morning of the experiment.	**Primary:** Exercise time. **Secondary:** Power output (W). Heart rate. RPE.	**18°C:** Exercise time: →. Power output: →. Heart rate: →. RPE: →. **30°C:** Exercise time: ↓ (9% faster). Power output: ↑. Heart rate: ↑. RPE: →.
Goekint et al. (33)	*N* = 11	60-min cycling at 55% of the maximal power output (Wmax) followed by a time trial until the participants completed a predetermined amount of work (equal to about 30 min cycling at 75% Wmax as fast as possible).	Reboxetine 16 mg (2 × 8 mg).	The night before and the morning of experiment.	**Secondary:** Exercise time. Heart rate. RPE.	Exercise time: ↑ (14.6 ± 15.5% slower). Heart rate: ↑ during 60 min cycling; → during time trial. RPE: →.
Klass et al. (34)	*N* = 10	60-min cycling at 55% of (Wmax) followed by a time trial until the participants completed a predetermined amount of work (equal to about 30 min cycling at 75% Wmax as fast as possible). Neuromuscular measurements of knee extensors (rectus femoris, vastus medialis, and biceps femoris).	Reboxetine 16 mg (2 × 8 mg).	The night before and on arrival in the lab on experiment day.	**Primary:** Exercise time. **Secondary:** Heart rate. RPE. Mean power output. MVC torque.	Exercise time: ↑ (9.4% slower). Heart rate: →. RPE: →. Mean power output: ↓. MVC torque: →.
Klass et al. (35)	*N* = 9	Repeated 3-s submaximal isometric contractions of the knee extensors (rectus femoris, vastus medialis, vastus lateralis, and biceps femoris) with a 2-s rest between each contraction and performed until task failure.	Reboxetine 16 mg (2 × 8 mg).	The night before and on the arrival in the lab on experiment day.	**Primary:** Endurance time. **Secondary:** Heart rate. RPE. MVC torque.	Endurance time: ↓ (15.6% shorter). Heart rate: ↑. RPE: ↑. MVC torque: ↓ (41.1%).
Piacentini et al. (36)	*N* = 7	Cycling tests (time trial) starting at 65% Wmax until the participants completed a predetermined amount of work (equal to about 90 min cycling at 65% Wmax).	Reboxetine 8 mg (2 × 4 mg).	The night before and the morning of the experiment.	**Primary:** Exercise time. **Secondary:** Heart rate. Blood lactate. RPE.	Exercise time: →. Heart rate: →. Blood lactate: →. RPE: →.
Roelands et al. (37)	*N* = 9	60-min cycling at 55% of (Wmax) followed by a time trial until the participants completed a predetermined amount of work (equal to about 30 min cycling at 75% Wmax as fast as possible) in temperate (18°C) or warm (30°C) conditions.	Reboxetine 16 mg (2 × 8 mg).	The night before and the morning of the experiment.	**Primary:** Exercise time. **Secondary:** Power output. Heart rate. Blood lactate. RPE.	**18°C/30°C:** Exercise time: ↓. Power output: ↓. Heart rate: ↑. in 18°C; → in 32°C. Blood lactate: ↓ in 18°C. RPE: →.
Meeusen et al. (38)	*N* = 7	Cycling to exhaustion at 65% Wmax.	Ritanserin (0,3 mg/kg).	Two capsules 24 h the day before and immediately before the experiments.	**Primary:** Time to exhaustion. **Secondary:** Heart rate. Blood lactate. Respiratory quotient (RQ).	Time to exhaustion: →. Heart rate: →. Blood lactate: →. RQ: ↑.

N, total sample size; MVC, maximal voluntary contraction; RPE, rating of perceived exertion; →, no changes; ↑, increase/longer time; ↓, decrease/shorter time.

**TABLE 3:** Antipsychotics: experimental testing, drug and measured performance parameters.

References	Sample size	Experimental testing	Drug	Time of drug intake	Performance and physical parameters (*primary/secondary*)	Statistical outcome (*data compared to placebo/all trials*)
Adamson et al. (17)	*N* = 5	Visual stimulus test. Grip strength test. Lumbar pull. Chinnings (pull-ups).	Oxypertine 0 mg/5 mg/10 mg/20 mg/40 mg.	Each dose repeated twice 2 h before starting the tests.	**Secondary:** Reaction time. Grip strength. Lumbar pull. Chinning number.	Reaction time: →. Grip strength: ↓. Lumbar pull: ↓. Chinning number: →.
Kim et al. (39)	*N* = 30 HC = 15	Incremental cycling (start at 30 W with an increase of 10 W per minute) until exhaustion.	Clozapine: participants treated were divided into groups based on their antipsychotic medication status: those treated mainly with clozapine (i.e., in an amount greater than 50% of total CPZE) comprised the clozapine group, those treated mainly with other antipsychotics (i.e., in an amount greater than 50% of total CPZE) comprised the non-clozapine group.		**Primary:** Resting heart rate. Peak heart rate. Heart rate reserve. **Secondary:** VO_2_ peak. Oxygen pulse. Ventilation. Respiratory exchange ratio. RPE.	Resting heart rate: ↑. Peak heart rate: ↑. Heart rate reserve: ↓. VO_2_ peak: ↓. Oxygen pulse: →. Respiratory exchange ratio: →. RPE: → (*compared to the non-clozapine group).*
Perez-Cruzado et al. (40)	*N* = 62	Physical fitness test battery: – Passive knee extension test. – Calf muscle flexibility test. – Anterior hip flexibility test. – Functional shoulder rotation test. – Timed-stand test. – Partial sit-up test. – Seated push-up test. – Grip test. – Single leg stance (open and closed eyes). – Functional reach test. – 2-min step test.	Risperidone consumer. Olanzapine consumer.		**Secondary:** Physical fitness: Aerobic condition. Flexibility. Balance. Strength.	**Risperidone:** Flexibility: →. Balance: →. Strength: →. Aerobic condition: ↓ (*compared to the non-risperidone-group).* **Olanzapine:** Flexibility: →. Balance: ↓. Strength: ↓. Aerobic condition: ↓ (*compared to the non-olanzapine-group).*

N, total sample size; RPE, rating of perceived exertion; →, no changes; ↑, increase/longer time; ↓, decrease/shorter time; HC, healthy control.

**TABLE 4:** Sedatives: experimental testing, drug and measured performance parameters.

References	Sample size	Exercise testing	Drug	Time of drug intake	Performance and physical parameters (*primary/secondary*)	Statistical outcome (*data compared to placebo/all trials*)
Charles et al. (18)	*N* = 8 + 27	Study 1: cycling 10 min at 120 Watts, than until to exhaustion with the work load being increased by 20 watts each minute. Study 2: cycling for 4 min at 50, 100, and 150 watts, respectively, and then until exhaustion.	Nitrazepam 10 mg. Temazepam 30 mg.	Double-Dummy protocol for 9 nights: Period 1: Temazepam (3 × 10 mg). Period 2: Nitrazepam (2 × 5 mg). Period 3: placebo 2 weeks interval between each period. Medication was taken 0.5 h before retiring.	**Secondary:** Peak level of exercise. Heart rate. Mean oxygen (O_2_) consumption.	**Study 1:** **Nitrazepam** Peak level of exercise: ↓. Heart rate: →. Mean O_2_ consumption from 140 W: ↑. **Temazepam:** Peak level of exercise: ↓. Heart rate: →. Mean O_2_ consumption: →. **Study 2:** **Nitrazepam:** Peak level of exercise: ↓. Heart rate: ↑. Mean O_2_ consumption: →. **Temazepam:** Peak level of exercise: ↓. Heart rate: ↑. Mean O_2_ consumption: →.
Collomp et al. (41)	*N* = 7	Wingate Test on a cycle ergometer	Lorazepam 1 mg	4 h before exercise testing	**Primary:** Peak power (PP) **Secondary:** Mean power (MP). Percentage of power decrease (% PD). Blood lactate.	Peak power: ↓. Mean power: →. % PD: →. Blood lactate: ↓.
Collomp et al. (42)	*N* = 7	Cycling at 85% VO2max until exhaustion.	Lorazepam 1.5 mg.	3 h before exercise testing.	**Primary:** Exercise time. **Secondary**: Blood lactate.	Exercise time: →. Blood lactate: ↓.
Ergen et al. (43)	*N* = 24	6× distance shooting (each round 3 arrows in 8 series, means in summary 24 shots).	Diazepam 5 mg.	Not provided.	**Primary:** Shooting scores. **Secondary:** Heart rate. Clicker reaction time. Aiming behavior. Center of pressure.	Shooting scores: →. Resting heart rate: →. Shooting heart rate: →. Clicker reaction time: →. Aiming behavior: →. Center of pressure: →.
Grobler et al. (44)	*N* = 12	30-m sprint test. Agility test. Graded treadmill running test to exhaustion.	Loprazolam 2 mg. Zopiclone 7.5 mg.	10 pm the night before exercise testing.	**Secondary:** Sprint time. Total time agility test. Time to exhaustion. Oxygen uptake. Max. ventilation rate. Max. heart rate. RPE.	**Both drugs:** Sprint time: →. Total time agility test: →. Time to exhaustion: →. Oxygen uptake: →. Max. ventilation rate: →. Max. heart rate: →. RPE: →.
Ito et al. (8)	*N* = 8	Vertical jumps. 50-m sprint.	Zolpidem 10 mg	11 pm the night before exercise testing.	**Secondary:** Vertical jumps (cm). 50-m sprint (s).	Vertical jumps: →. 50-m sprint: →.
Ito et al. (46)	*N* = 21	Forward bending. Right grip strength. Right quadriceps muscle strength. Repeated side jumps.	Zaleplon 10 mg.	Immediately before going to bed.	**Secondary:** Forward bending (cm). Grip strength (kg). Quadriceps strength (kg). Repeated side jumps (n).	Forward bending: →. Grip strength: →. Quadriceps strength: →. Repeated side jumps: →.
Suda et al. (45)	*N* = 12	Vertical jumps. 50-m sprint. Repeated side jumps.	Eszopiclone 2 mg.	Immediately before going to bed (11.00 pm).	**Secondary**: Vertical jumps (cm). 50-m sprint (s). Repeated side jumps (n).	Vertical jumps: →. 50-m-sprint: →. Repeated side jumps: →.
Tafti et al. (9)	*N* = 8	Standing jump test. Running time test.	Zopiclone 7.5 mg.	11 pm the night before exercise testing.	**Secondary:** Standing jump. Running time.	Standing jump: →. Running time: →.

N, total sample size; RPE, rating of perceived exertion; →, no changes; ↑, increase/longer time; ↓, decrease/shorter time.

**TABLE 5:** Stimulants: experimental testing, drug and measured performance parameters.

References	Sample size	Experimental testing	Drug	Time of drug intake	Performance and physical parameters (*primary/secondary*)	Statistical outcome (*data compared to placebo/all trials*)
King et al. (47)	*N* = 15	Muscle-fatiguing handgrip task during functional magnetic resonance imaging.	Methylphenidate 20 mg.	Before the start of the exercise testing	**Primary:** Mean grip force.	Mean grip force: ↑.
Klass et al. (34)	*N* = 10	60-min cycling at 55% of (Wmax) followed by a time trial until the participants completed a predetermined amount of work (equal to about 30 min cycling at 75% Wmax as fast as possible). Neuromuscular measurements of knee extensors (rectus femoris, vastus medialis, and biceps femoris).	Methylphenidate 40 mg.	On arrival in the lab on experiment day.	**Primary:** Exercise time. **Secondary:** Heart rate. RPE. Mean power output. MVC torque.	Exercise time: →. Mean power output: →. Heart rate: ↑. RPE: →. MVC torque: ↓.
Roelands et al. (48)	*N* = 8	60-min cycling at 55% of Wmax followed by a time trial until the participants completed a predetermined amount of work (equal to about 30 min cycling at 75% Wmax as fast as possible) in temperate (18°C) or warm (30°C) conditions.	Methylphenidate 20 mg.	1 h before the start of exercise.	**Primary:** Exercise time. **Secondary:** Power output. Heart rate. RPE.	**18°C:** Exercise time: →. Heart rate: →. RPE: →. **30°C:** Exercise time: ↓ (16% faster). Power output: ↑. Heart rate: ↑. RPE: →.
Westover et al. (49)	*N* = 245	Treadmill exercise test until exhaustion.	An amphetamine- or methylphenidate-type (AMP/MPH) stimulant.	Dosage and duration of medication use was not provided.	**Primary:** Peak heart rate. **Secondary:** Peak systolic blood pressure (SBP). Average rise in systolic blood pressure. Estimated VO_2_max.	Peak heart rate: ↓. Peak SBP: →. Average SBP: →. Estimated VO_2_max: →.

N, total sample size; MVC, maximal voluntary contraction; RPE, rating of perceived exertion; →, no changes; ↑, increase/longer time; ↓, decrease/shorter time.

#### Antidepressants

Our search resulted in 21 studies that met our inclusion criteria. The majority examined the effects of antidepressants (fluoxetine, paroxetine, bupropion, and reboxetine) on physical/athletic performance, which were measured through ergometric cycling or isokinetic muscle measurements. There were 17/21 studies for antidepressants, that only recruited male participants with similar age (approximately between 19 and 27 years old). There is only one study with female and just 2/21 studies with mixed participants.

##### Effects of paroxetine

Our search resulted in six publications examining paroxetine (21–26). All drug administration of paroxetine were given as a single-dose of 20 mg in physically active men (21–26) and women (21, 25). In addition, one study further compared 10 and 40 mg of paroxetine but they did not affect physical performance (24). Another study showed that paroxetine did not influence exercise duration of cycling as well (22). Three other studies found that paroxetine decreased physical performance in cycling (23, 24, 26). Paroxetine increased activation of unfatigued muscle but exacerbated central fatigue during prolonged sustained contractions (21). During a sustained submaximal contraction, paroxetine had no influence on motor performance or on the development of central fatigue (25). Heart rate and lactate blood concentration increased during exercise but did not differ between trials (22–24, 26).

##### Effects of fluoxetine

Exercise performance during cycling was not affected by fluoxetine (20 mg) in well-trained young men (27). Another RCT examined that there were no significant effects of fluoxetine on strength or high-intensity exercise performance in young male athletes and no parallel change in muscle strength of knee extensors was measured after acute fluoxetine administration (40 mg, 6 h before testing) (19). There was no suppression of maximal voluntary contraction strength with continuous fluoxetine administration (40 mg, every day for 2 weeks) (19). Blood lactate concentration during exercise testing was only measured in one study (27). Lactate increased throughout exercise but was not influenced by fluoxetine. Another study examined further SSRI and showed that the isokinetic muscle performance of depressed middle-aged patients (m/w) was improved after a 3-month treatment with different SSRI (fluoxetine, sertraline, fluvoxamine, or citalopram) (28).

##### Effects of bupropion

Six publications examined the effects of bupropion (6, 20, 29–32). One clinical trial showed that the acute administration of a single dose of bupropion (2 × 300 mg) during prolonged exercise cycling in a temperate conditions had no effect on performance in well-trained male cyclists (31). But in warm conditions (30°), acute bupropion administration enabled endurance-trained males to 9% faster completed time trials (cycling) in the bupropion condition compared to placebo (6). Two further trials supported this finding, both in physically active women (29) and in well-trained male cyclists (32). Continuous administration of bupropion (10 days of medication) caused no differences in cycling performance in trained males (20). Neuromuscular data showed that maximal voluntary contraction and voluntary activation were unaffected by the administration of bupropion (2 × 300 mg) during cycling in temperature (20°) and warm (32°) conditions in physically active males (30). Heart rates increased over time during exercise cycling (6, 20, 31, 32) and exercise intensity (30), but were not influenced by drug administration. The results of Cordery et al. (29) showed significant changes in heart rate, but only at the end of the exercise test. Blood lactate concentration was not influenced by bupropion (31). The subjects of all six studies were of similar age (mid-late 20s).

##### Effects of reboxetine

We identified five randomized controlled trials with reboxetine (33–37). All drug administration of reboxetine were given as a single-dose, examined in young physically active males. One study showed no differences in cycling performance between reboxetine (8 mg) intake and placebo (36). Nearly all other studies showed decreases in exercise performance after reboxetine (16 mg) (33–35, 37). Lactate blood concentration (36) and heart rate (33, 36) changed but were not influenced by reboxetine intake. But in one study, heart rate was significantly higher in the reboxetine condition compared to placebo in cooler environment (18°) (37). This drug-induced effect could not be replicated in the heat (30°) (37). Blood pressure was not influenced by drug. At the end of the time trials and after recovery in reboxetine (18°), the blood lactate concentrations were significantly lower than during placebo (18°) (37).

##### Effects of a further antidepressant drug

The results of one study showed that a specific centrally acting 5-HT_2*A/*2*C*_ antagonist (5-Hydroxytryptamin/Serotonin, single-dose Ritanserin, 0,3 mg/kg) did not influenced the time to exhaustion in cycling in trained males (38). Respiratory quotient at the end of exercise was significantly higher in Ritanserin group, while the increase during exercise was comparable in all groups (38).

#### Antipsychotics

Our search resulted in three publications using the antipsychotic drugs oxypertine (17) (rarely used in clinical practice), clozapine (39), olanzapine and risperidone (40). There are 2/3 studies that recruited mixed participant and 1/3 study was only with male participants. In all studies the subjects were approximately between 20 and 30 years old. Oxypertine was examined in different single-doses from 0 to 40 mg. Physical performance in several exercise tests (muscular force, muscular endurance) in trained athletes (gender unknown) declined progressively as the dosage was increased (17). Negative effects were also found with olanzapine and risperidone (no single-dose) in several physical exercise tests (flexibility, balance, strength, and aerobic condition) examined in a cross-sectional study with middle-aged patients (m/w) (40). Clozapine treatment (no single-dose) was associated with reduced cycling performance in middle-aged patients (m/w) with schizophrenia or schizoaffective disorder compared with non-clozapine antipsychotics (39).

#### Sedatives

Our search resulted in nine studies using benzodiazepines (18, 41–44) and z-drugs (8, 9, 44–46). For sedatives there are 4/7 studies that recruited mixed participants and 3/7 were only with males. In all studies the subjects were approximately between 18 and 26 years old.

##### Benzodiazepine

One study applied temazepam 30 mg, nitrazepam 10 mg and placebo for 9 days in young university students (m/w) (18). On day 2, maximal attained exercise levels in cycling with temazepam 30 mg and nitrazepam 10 mg were comparable to placebo, on day 9, physical performance with nitrazepam was lower than with temazepam and placebo (18). One study showed that 4 h after drug intake, a single-dose of lorazepam 1 mg impaired anaerobic peak power and induced a significant decrease in blood lactate concentration during a Wingate test in healthy volunteers (m/w) (41), while another study found that 3 h after a single-dose of lorazepam 1.5 mg time of cycling was not significantly changed in young male triathletes (42). Diazepam 5 mg (exact time of intake was not provided) was shown to not significantly change shooting performance in Turkish archers (m/w) (43). Ten hours after night-time administration, a further study showed no significant impairment of physical performance in sprint, agility and running after a single-dose of loprazolam 2 mg, but drug intake resulted in a greater hangover effect physically active volunteers (m/w) (44).

##### Z-drugs

Five further studies examined the effects of z-drugs on physical performance with no significant adverse effects on performance. Two RCTs could not identify impaired athletic performance measured with a vertical jump and a 50-m sprint after taking zolpidem 10 mg (8), as well as no increased endurance time after a running test with zopiclone 7,5 mg (9), both with two doses over two nights in young male athletes. As well, 10 h after night-time administration of single-dose zopiclone 7.5 mg physical performance in sprint, agility and running was not significantly impaired in physically active volunteers (m/w) (44). A recent study assessed the residual effects of a single-dose eszopiclone 2 mg on physical performance in male athletes and resulted in no significant differences in vertical jump, 50-m sprint and repeated side jumps (45), as well as a single-dose of zaleplon 10 mg that did not affect physical performances in forward bending, grip strength, quadriceps strength and side jumps (46).

#### Stimulants

For stimulants there are 2/4 studies that recruited mixed participants and 2/4 were only with males. In all studies the subjects were approximately between 26 and 42 years old. In contrast to sedatives, stimulants could possibly enhance physical performance. This hypothesis was supported by the results of two studies with acute administration of methylphenidate 20 mg after a handgrip task (in young healthy volunteers m/w) and cycling (in young well-trained males) (47, 48), while one study revealed no significant changes in cycling performance after single-dose methylphenidate 40 mg in well-trained males (34). A cross-sectional study examined stimulant medication use and maximal exercise running test outcomes in a large community sample (49). Stimulant medication use was not associated with increases in peak systolic blood pressure (SBP) and average SBP rise, nor did it impact cardiorespiratory fitness (VO_2_max). Dosage and duration of use of medications were not available (49). Heart rate significantly increased by methylphenidate in warm condition (48) and was higher at the end of the time trial for methylphenidate than for the placebo (34). But it was also shown, that stimulant medication users had a significantly lower peak heart rate (49).

## Discussion

With 33 included studies, this is the first systematic review examining the effects of psychiatric medication on physical performance in physically healthy adult humans. Differentiated by their medical compound, dose and period of use, psychotropic drugs can have performance-limiting or performance-enhancing effects. These findings can help to provide further information about the medication to the patients and thereby lead to reduced discontinuation rates. In the following, we discuss four medication groups separately (antidepressants, antipsychotics, sedatives, and stimulants).

### Antidepressants

Within the antidepressants, nine studies have been conducted examining the effects of SSRI on exercise performance using paroxetine (21–26) and fluoxetine (19, 27). Three studies (22, 23, 26) with comparable dosages and application times of paroxetine led to contradictory conclusions. Two studies (23, 26) showed reduced total exercise time, one did not observe any difference after the intake of paroxetine (22). In contrast, another study (22) using the same dose and same time of administration did not observe any difference in total exercise time. The major difference in the study protocols and therefore a possible explanation for the different findings was the manipulation of the ambient temperature (30°C) (22) at which the exercise was performed compared to that of the previous studies (23, 26). It has been suggested that serotonin may play a role in thermal regulation and elevated body temperature is a major factor in the development of fatigue during prolonged exercise (50). The results suggest that oral administration of 20 mg paroxetine did not significantly influence the thermoregulatory factors that may limit exercise capacity (22). Based on these results, the authors of a later published study (24) hypothesized that the dose of 20 mg paroxetine might be insufficient to consistently influence the serotonergic system and consequently physical performance. In their study, they tested doses ranging from 10 to 40 mg of paroxetine. Surprisingly, the higher dose of 40 mg did not affect physical performance. Physical performance only decreased after administration of 20 mg of paroxetine (24). The authors explained this difference based on the pharmacological profile of this agent. Paroxetine increases the extracellular concentration of brain 5-HT, which can promote the release of dopamine (51). A higher dose of 40 mg paroxetine may result in a higher stimulation of 5-HT3 receptors compared to 20 mg of paroxetine, thus increasing dopamine release into the synaptic cleft (24). Furthermore, the authors assumed that 5-HT receptor responses could be influenced by an individual’s aerobic capacity level. They suggest that individuals with higher aerobic capacity would be less affected by psychiatric medication than those with a low aerobic capacity level. However, their results revealed decreased physical performance in the high aerobic capacity group which suggested that individuals with higher aerobic capacity might be more responsive to pharmacological activation of the serotonergic system during exercise (24). The authors discussed various explanations for this observed phenomenon, such as higher body fat content in volunteers with lower aerobic capacity, differences in thermoregulation or changes in carbohydrate availability or acid-basic balance between the groups, but they could not postulate a ‘one-size-fits-all’ hypothesis (24).

Two studies observed no impact of fluoxetine on physical performance, neither after acute (19, 27) nor after continuous drug administration (19). The authors of another study grappled with the *central fatigue hypothesis*, which states that an increased concentration of brain 5-HT as a result of a long-term exercise may contribute to the perception of central fatigue. However, their results showed that subjects were not hindered by the central fatigue hypothesis during the 80 and 90% VO_2_max trials (19) also not in the previously mentioned study during 65% Wmax (27). This can possibly be attributed to peripheral mechanisms that contribute to fatigue during short duration exercise training (52). A previously published study showed that, at a lower intensity, there is an earlier onset of fatigue during 70% VO_2_max cycle ergometer test, after the administration of a SSRI (paroxetine) (26). Similarly, subjects expressed a greater perceived exertion during a cycle ergometer test after receiving fluoxetine (5). Both of the cited studies indicated that SSRI administration may play a role in fatigue during endurance exercise. Interestingly, the results of the chronic study (cycling at 90% VO_2_max) showed reduced ventilation (7.5%) during the fluoxetine trial with no alteration in performance (19). The authors discussed that this may have been caused by inhibitory and excitatory 5-HT receptors that have been identified in the respiratory system of the cat (53). The role of the 5-HT metabolism during exercise is very complex which was also discussed in Kavanagh et al. (21) and Thorstensen et al. (25). The studies showed that with increased 5-HT availability voluntary muscle activation and torque generation increased during unfatigued maximal contraction and in contrast, the ability to generate maximal torque was compromised under fatigue conditions (21). The authors suggested that serotonergic drive, that occurred with prolonged maximal contractions, provided a spinal mechanism by which higher concentrations of 5-HT may contribute to central fatigue (21). Interestingly, during sustained submaximal contractions, enhanced availability of 5-HT did not directly influence motor performance (25). Only strong fatiguing contractions causing strong serotonergic drive may cause 5-HT-associated reductions in motor output. It should be further studied to understand its mechanisms of action during exercise and especially its role in the onset of fatigue. However, the serotonergic system (could potentially) impact fatigue during exercise, but the mechanisms underlying this relationship remain elusive. Of the mentioned studies investigating how 5-HT concentration affects exercise performance, most of them used well-trained/physically active subjects (19, 22–24, 26, 27). In fact, only one publication included patients in their study. Its results showed that isokinetic muscle performance in patients with major depressive disorder was reduced compared to healthy controls. After 3 months of antidepressive treatment (comprising a trial of fluoxetine), muscle performance improved and the patients approached levels similar to the healthy control group. The authors suggested that this may be related to an increasing metabolism in brain structures, such as prefrontal cortex and basal ganglia (28). Although two other studies identified SSRI-mediated changes in central fatigue during sustained isometric contraction, their participants had no training history (21, 25), SSRI responses may be associated with the type of exercise as well as the individual’s fitness level which was discussed earlier (24). In conclusion, it can be stated that the here presented studies have mostly shown limitations in physical performance. The explanations for the mentioned impairments may relate to the modification of central fatigue, which has not been adequately investigated so far. Moreover, there is no data on the nowadays commonly used preparations citalopram, escitalopram and sertraline.

With its norepinephrine dopamine reuptake inhibitor function, bupropion does not typically cause weight gain or sedation (54). The included studies suggest that there may be performance enhancement in heat (6, 29) but not in temperate conditions (31). Similar performance and thermoregulatory effects did not occur after continuous administration of bupropion over several days, which might be due to an adaptation of central neurotransmitter homeostasis (20). Further examinations (32) investigated a dose-response relationship. The study demonstrated that an ergogenic effect occurred when the highest dose (600 mg) of bupropion was taken, which is consistent to prior findings (6). Bupropion was shown to cause a suppressed sensation of heat enabling humans to push themselves to higher temperature without perceiving greater effort (6). This may potentially increase the risk of developing heat illness (6). Because of its ergogenic effect, which can lead to enhanced performance, it is included in the Monitoring Program of WADA (World Anti-Doping Agency) competition but is not prohibited in competition (54).

Similar examinations have been conducted with the norepinephrine reuptake inhibitor reboxetine. One study (36) found no effect of reboxetine 8 mg on performance (36). In contrast, two other studies showed that this medication may limit performance (33, 37). To identify the mechanisms that contributed to the decrease in performance after reboxetine 16 mg (37), other researchers analyzed the potential link between neural impairments and changes in brain neurotransmission (34, 35). Their results suggest that noradrenaline reuptake inhibition could contribute to the development of supraspinal fatigue observed after a prolonged cycling exercise (34) and after a fatiguing task involving intermittent submaximal contractions (35). Since corticospinal excitability was not modified after both exercising tests, norepinephrine appears to more specifically affect supraspinal centers located prior to the motor cortex.

In conclusion, the studies of antidepressants on physical performance must be considered heterogeneous and thus the observed effects inconsistent. Possible reasons for this include differences in study protocols (doses and acute versus continuous intake of the medication) and limitations (e.g., usually very small sample sizes). Only bupropion 600 mg resulted in performance enhancement in heat (6, 29, 32) but not in temperate conditions (31) with risk of contracting heat illnesses. Other antidepressants (paroxetine, fluoxetine, and reboxetine) led to impairments or showed no medication-induced changes during exercise. The mentioned drugs are usually not related to sedation. Therefore, further research is needed to elucidate the effects of psychotropic drugs that can cause greater sedation (e.g., mirtazapine or tricyclic antidepressants).

### Antipsychotics

The effects of antipsychotics are more consistent and point to limited physical performance, even though only three studies could be included in this review (17, 39, 40). One study showed dose-dependent effects, with a gradual impairment as the dose level of oxypertine was increased from 5 to 40 mg (17). Another study showed that olanzapine had negative effects on muscle strength, balance and aerobic endurance and risperidone had negative effects on the amount of physical activity (40). The authors assumed that patients with severe psychiatric symptoms have a decrease in their activity levels and physical fitness in general. These facts may also be explained by side-effects such as increased fatigue or sedation, both of which might affect physical performance (40). These data confirm previous findings from a study that was not included in our review (different population), but showed that adults with severe mental illness (SMI) who were taking antipsychotic medication were less physically active and had lower body balance as well as muscular strength compared with unmedicated patients (12). Another study from our review showed that cardiovascular fitness was impaired during clozapine treatment which might be explained by the higher antagonistic activity at alpha-adrenergic receptors. This antagonism could lead to limited blood flow in the working skeletal muscle and compromise cardiovascular stability during exercise (39). We found no articles on the use of antipsychotic medication in athletes but based on the assumptions from the cited studies (12, 40), this collective might expect less limitations compared to those previously mentioned in patients. Due to a favorable side-effect profile, aripiprazole or lurasidone are recommended for this special collective (2). In addition, an important difference to the results from the antidepressants should be emphasized. The presented studies using antipsychotics have mostly included patients with a mental illness who have been taking the medication for a longer time period (39, 40). This may explain the consistent deterioration in physical performance with antipsychotic treatment. For antidepressants, mostly acute administration and well-trained subjects were investigated.

### Sedatives

Sedatives are mostly used in clinical practice to treat insomnia and other sleep disturbances (55). They are a common issue for athletes after jet lag or as isolated symptoms in stressful situations. Commonly used sleep aids among the general population include benzodiazepines and z-drugs.

It would seem obvious that the intake of sedating medication at night may result in residual morning drowsiness, which may impair the participant’s motivation and may increase perceived exertion. However, these assumptions were not confirmed by the identified studies in our review. The intake of loprazolam (2 mg) and zopiclone (7.5 mg) at 10 pm the night before the exercise test resulted in unchanged physical performance (44). The authors suggested that zopiclone may have a weaker (self-reported) hangover effect compared to loprazolam, but both drugs did not significantly impair objectively measured physical performance (44). A greater number of subjects reported feeling alert after the ingestion of zopiclone without having any residual drowsiness the morning following the night-time administration. This can possibly be attributed to the different half-lives of zopiclone [4–5 h, (56)] and loprazolam [6–12 h, (57)]. Previously published studies that were not included in our review (missing data about physical fitness) supported that finding and stated that due to the relatively short half-life of zopiclone, the hangover effect the morning after night-time administration was minimal (56) and a 7.5 mg dose of zopiclone has been shown to have the optimum sedative properties with negligible side-effects (58). Similar effects are shown with three further ‘z’ agents that were used in double-doses but showed no adverse effect on physical performance (8, 9, 46). This can possibly be explained by their short plasma half-life and limited duration of action (59). Zaleplon is characterized by an ultrashort half-life (approximately 1 h). Zolpidem and zopiclone have longer half-lives (1.5–2.4 h and 4–5 h) (59). These properties, together with the low risk of residual effect, may explain the limited negative influences on physical performance (59). Instead, the authors concluded that zopiclone and zolpidem have useful hypnotic activity without disturbing physical performance on the following day (8, 9), suggesting that zolpidem may be used in healthy humans to adjust sleep disturbances (8).

Similar results were shown by another study evaluating psychomotor and physical performances on the following day after taking eszopiclone (half-life 5–6 h) at bedtime (45). The authors did not identify any impairment. Another study examined the longer acting benzodiazepines temazepam [half-life of 5.3 h (60)] and nitrazepam [half-life of 30 h (61)] and their effects on exercise performance using two separate study designs (study 1: drug for two nights, study 2: drugs for nine nights) with night-time administration (18). In study 1, at all work-loads both drugs showed an increase in ventilation, gas exchange and heart rate when compared to placebo suggesting that the subjects were working harder at all work loads and this might have reduced the maximum load they could achieve. In study 2, a similar trend in the maximal exercise load was displayed with a significant difference in the nitrazepam group on day 9 compared to temazepam and placebo. The authors attributed this finding to a probable hangover effect of nitrazepam (18).

Two further studies investigated the effects of benzodiazepines on physical performance (41, 42). Subjects who had been treated with lorazepam (1.5 mg) 4 h before cycling at 85% of their VO_2_max (submaximal exercise) showed no difference when compared to placebo but exhibited significantly lower values in selected hormones and lactate (42). On the other hand, subjects treated with lorazepam (1.0 mg) 3 h before exercising a Wingate Test (supramaximal exercise) showed significantly lower values in peak power and maximal lactate when compared to placebo (41). Authors in both studies suggested that the lower lactate values may be due to either a decrease in muscle glycogenolysis or a change in lactate removal (41, 42) but in Collomp et al. they did not find a clear association between drug effects and impairment in peak power (41). Melatonin is one of the most studied sleep aids among athletes (55), but no study met our inclusion criteria due to an inappropriate study design. However, one study indicated no impairment of performance the following day after intake (62).

In summary, due to the heterogeneous study designs (drug, dosage, and exercise testing) of the studies included in our review, it is difficult to derive consistent findings about sedatives and their effects on physical performance. Sedatives may result in residual morning drowsiness, which may impair the participant’s motivation but not directly physical performance which may be explained by different half-lives. Especially with regard to their use in sleep disorders or stage fright, sedatives should be further investigated, since reduced muscle strength and poorer running times in the endurance sport have already been reported (63).

### Stimulants

Stimulants are known to be performance-enhancing and they are banned (only) in competition (64). This was first reported in the Journal of the American Medical Association (JAMA) (65). Since then, further studies have reported similar effects with performance enhancements in anaerobic capacity, strength, time to exhaustion, acceleration, and maximum heart rate (66). Some researchers suggest that athletes taking stimulants (e.g., methylphenidate) may be able to exercise to higher core temperature without any change in the perception of effort or thermal stress. The ergogenic effect was confirmed by one of our included studies (48). Subjects showed improved performance in the heat but not in temperate conditions without any greater perception of effort or thermal stress. Concerning this matter, stimulants might not only be ergogenic but also harmful in the heat as athletes may be unaware of increasing thermal/heat stress promoting heat illness (6, 48).

In another experiment, subjects demonstrated enhanced force and changes in brain connectivity throughout a muscle-fatiguing handgrip test using acute ingestion of 20 mg methylphenidate at room temperature (47). The authors discussed, that given agents may affect dopamine transmission and consequently cause an increase in fatigue and a decrease in motivation (67), methylphenidates influence on neurotransmission make subjects more willing to exert forces closer to their maximum (68). The ergogenic mechanism of methylphenidate remained elusive in this study, further experiments were proposed to detect the neurochemical underpinnings of motivation and muscle fatigue.

One RCT tested the effects of acute noradrenaline (reboxetine 8 mg) and acute dopamine (methylphenidate 40 mg) reuptake inhibitors on prolonged cycling exercise and supraspinal fatigue in a temperate environment (34). Compared to reboxetine, methylphenidate did not affect exercise performance and did not contribute to the development of supraspinal fatigue. It can be speculated that compared to dopamine, norepinephrine has an inhibitory effect on performance. The effects of reboxetine have already been discussed in the paragraph regarding antidepressants (34).

We were further able to identify a cross-sectional study examining stimulant medication use and maximal exercise test outcomes in a large community sample (49). In contrast to previous studies that have shown an increase in heart rate associated with acute application of methylphenidate (34, 48), this study showed a lower peak heart rate in stimulant medication users compared to non-users (49). The reasons were unclear, however, authors suggested that unmeasured confounding of stimulant use or confounding by contraindication and survivor bias caused lower peak heart rates.

## Conclusion

This is the first systematic review evaluating the effects of psychiatric medication on physical performance. We identified studies with antidepressants, antipsychotics, sedatives and stimulants. Antipsychotics seemed to be performance impairing, while the findings for antidepressants and sedatives were more inconsistent. Stimulants were the only group with consistent performance-enhancing effects.

Several issues impede the formulation of generalized conclusions for treatment regimes and should therefore be further considered in future studies. Most studies were conducted in populations of athletes, limiting general transferability of the results. Although 27 of our included studies have powered their sample size calculation to the primary sport outcome, most of them were conducted in small sample sizes (*N* < 10). To get an impression of sample sizes for future studies, we made some assumption using G*Power (69). Assuming a two-sided independent *t*-test for a respective analysis, a power of 0.8 and an α-prolity of 0.05, a sample size of 34 subjects per group would be valid to identify a moderate effect size *d* = 0.5 and 19 subjects per group to identify a large effect size of *d* = 0.7 between two groups in a conservative between-group design. A within-group design using pared sample *t*-tests would results in slightly smaller sample sizes. Of our included studies five out of 36 included >26 subjects. Thus, most studies may have been underpowered and we recommend larger sample sizes for future studies. Moreover, most study protocols used single dose medication, which does not reflect real-world settings in clinical practice. Within the different medication groups, there was a bias toward specific drugs (e.g., six studies with paroxetine and none for the often-used venlafaxine). In addition, there were 17/21 studies for antidepressants, that only recruited male participants. This shows good comparability among these studies, since the age of the participants was almost identical, but there is only one study with female and just 2/21 studies with mixed participants. For antipsychotics, there are 2/3 studies, for sedatives there are 4/7 studies and for stimulants there are 2/4 studies that recruited mixed participants. However, future studies especially with antidepressants in female and mixed participants are necessary to be able to make sex-related conclusions and to state more details for the treatment reality.

Further research with longitudinal studies and therapeutic doses is recommended to focus on precisely these issues to be able to derive even more well-founded data and clinically reliable recommendations for therapy. We would suggest adding physical performance parameters such as e.g., exercise time, oxygen consumption, heart rate, muscle contraction or blood lactate concentration to the safety assessments in future clinical and approval studies for psychopharmacological compounds. On the one hand, one could assume that with an approval in psychopathology also physical performance parameters may improve. However, the here evaluated outcomes from mainly single-dose studies imply that a worsening of physical performance parameters need to be defined and evaluated as compound-specific side-effects. Moreover, with a deeper understanding of these effects of psychopharmacological compounds on physical performance parameters, medication prescriptions could become more tailored to individual requirements and addressed by specific side-management interventions. Less side effects due to impairments of physical performance (which might be misinterpreted as sedation) could lead to reduced discontinuation rates of treatment and therefore more successful treatments in terms of less discontinuation-based relapses.

## Author contributions

AHi, DL, and AR conceived the study and performed the qualitative analyses. AHi wrote the first draft of this manuscript. AR and AlH supervised the project. EW provided methodological advice. All authors were involved in reviewing the manuscript and approved the final version of the manuscript.
