# Supplementary material for: Psychiatric medication and physical performance parameters – Are there implications for treatment?
Source: Front Psychiatry. 2022 Sep 6;13:985983. doi: 10.3389/fpsyt.2022.985983 (PMC9488519; doi:10.3389/fpsyt.2022.985983)
Supplement: Supplementary file 1 [file Data_Sheet_1.docx]

Supplementary Material

# Relevant statistical outcomes of the included studies

| **Reference** | **Results/ Statistical outcome** |
| --- | --- |
| Adamson et al. (15) | There was a gradual impairment as the dose level was increased from 5 to 40 mg. Grip strength was 124.80 lb in 5 mg oxypertine over 118.13 lb in 40 mg oxypertine. Lumber pull was 648.83 lb in 5 mg oxypertine over 605.63 lb in 40 mg oxypertine. Muscular endurance and reaction time were unaffected. The subjects vary in their response to the different treatments, but the effect of the drug is not significant. |
| Bilici et al. (26) | The levels of peak torque and total work levels enhanced after antidepressant treatment, acceleration time significantly attenuated. Especially noticeable was total work in the left leg at 180° Extension before treatment compared to after treatment (855.43±401.66 vs. 1615.91±2074.92 J, t=4.45, df=22 p<0.01). Please refer to the original paper for the complete table. |
| Charles et al. (16) | The highest levels were always seen on placebo, with significance in the low value obtained with nitrazepam (246 W) in study 2 on day 9 (P<0.01) vs placebo (261 W) and vs temazepam (260 W). The mean oxygen consumption values from study in temazepam did not differ significantly from placebo at any level but the nitrazepam data were significantly higher at 140 watts and above (P<0.05, paired t-test). No significant differences were seen in study 2 up to the 150 watts levels. Highest mean heart rate was on temazepam and on day 9 with nitrazepam. |
| Collomp et al. (40) | Time of cycling was not significantly changed after lorazepam or placebo administration (22.9±2.5 vs. 23.5±3.8 min), it was increased in four subjects and decreased in three subjects. During the test, lactate concentrations with lorazepam were significantly lower at each time during exercise (P<0.05) compared with placebo. |
| Collomp et al. (39) | Lorazepam ingestion resulted in significant impairment in peak power (626±72 vs. 669±78 W) compared to placebo. Mean power and % of power decrease remained unchanged after lorazepam. Lactate at the end of exercise and at the maximum were significantly lower with lorazepam (end: 5.1±1.2 vs. 6.9±1.5 mmol, max: 9.5±1.5 vs. 12.4±1.8 mmol, P<0.05). |
| Cordery et al. (27) | There was an increase in performance of 7.5± 9.6% (P=0.042) in the bupropion. Work completed in each 5-min interval decreased throughout the exercise test (P=0.08) and was significantly different at 20 min compared with 5 min (P=0.023; d=0.523). Total work done was higher during the bupropion trial (291±48 kJ) than the placebo trial (269±46 kJ, P=0.042; d=0.468). Heart rate was higher at the end of the performance test on the bupropion trial (185±9 bpm) than on the placebo trial (179±13 bpm, P=0.043; d=0.537). |
| Ergen et al. (41) | Shooting scores did not show any significant difference between the measurements (F=2.09; p>0.05). Resting heart rate was 84.0±5.65 bpm and shooting heart rate was 113.1±12.2 bpm with diazepam. Clicker reaction time values were not significantly different in all conditions (F=0.23; p>0.05). There were no difference in aiming behavior (p>0.05). There were no difference in the velocity of center of pressure in x and y axis of the front and rear feet (p>0.05). |
| Goekint et al. (31) | Subjects performed significantly worse on the 75% time trial when on reboxetine treatment compared with the placebo treatment (P<0.01), with a mean increase of 14.6±15.5% for the time needed to complete the work output. During the time trial, no significant effect of drug intake on heart rate was revealed (P=0.23), but significant differences in heart rate were found between reboxetine and placebo treatment during the 60 min at 55% of Wmax (P=0.005). No difference was found between reboxetine and placebo treatment in RPE. |
| Grobler et al. (42) | No differences in the best sprint time between placebo vs. zoplicone vs. loprazolam (4.6±0.5 s vs. 4.7±0.5 s vs. 4.6±0.6 s). No difference in the best time taken to complete an agility test between placebo vs. zoplicone vs. loprazolam (12±1.5 s vs. 12±1.5 s vs. 12±1.6 s). No difference was found in the time to exhaustion (670±117 s vs. 680±95 s vs. 706±107 s), VO_2_ (4273±811ml vs. 4286±1003 ml vs. 4241±877 ml), ventilation (150±21 L vs. 145±28 L vs. 145±22 L) and max. heart rate (194±11 vs. 193±12 vs. 195±12 bpm) attained during the treadmill run following the ingestion of placebo, zopiclone, or loprazolam. |
| Ito et al. (8) | The vertical jumps and the 50-m sprint did not show any significant differences between zolpidem and placebo. The mean of vertical jumps was 61.05 cm in zolpidem and 60.70 cm in placebo, the mean of 50-m sprint was 7.44 s in zolpidem and 7.44 s in placebo. |
| Ito et al. (46) | There were no significant differences between zaleplon and placebo conditions in forward bending test (24.80±5.56 vs. 26.68±5.18 cm, P<0.86), right grip strength (42.30±1.46 vs. 40.88±1.40 kg, P<0.09), right quadriceps femoris muscle strength (50.50±2.36 vs. 49.24±2.76 kg, P<0.51), and repeated side jumps (53.97±1.33 vs. 54.15±1.10 no., P>0.72). |
| Kavanagh et al. (19) | Study 1: The maximal torque that was produced during the unfatigued MVCs was significantly greater for the paroxetine condition (66.2±26.1 Nm) compared to the placebo condition (63.2±23.4 Nm, t=3.237, P=0.006). Of the 14 participants, 11 generated greater maximal torque for the paroxetine condition (mean 5.5% increase) whereas 3 generated a lower maximal torque for the paroxetine condition (mean 2.7% decrease). Paroxetine caused greater sleepiness than placebo by the start of experimental testing. Significant effects of drug (F=9.147, P=0.012) were detected for rating of perceived fatigue. Study 2: The maximal torque that was produced during the unfatigued brief MVCs was significantly greater for the paroxetine condition (64.8±25.2 Nm) compared to the placebo condition (61.9±21.6 Nm, t=1.971, P=0.038). Voluntary activation was 1.5±1.8% higher for the paroxetine compared to the placebo condition in unfatigued contraction, but it was reduced for the paroxetine condition compared to the placebo condition (F=4.434, P=0.048) in fatigued contraction. Study 3: F-waves of the abductor digiti minimi (ADM) were unaffected by paroxetine for unfatigued muscle and marginally affected following a brief 2-s MVC. F-wave area and persistence were significantly decreased following a prolonged 60-s MVC of the ADM.  Following the 2-s MVC, F-wave area was reduced by 23.3±16.2% for the placebo condition and 27.3±19.4% for the paroxetine condition. F-waves decreased by 12.1±8.2% for the placebo and by 17.4±12.1% for paroxetine for the first minute following the 2-s MVC. F-wave persistence was reduced for the paroxetine condition compared to the placebo condition. Following the 60-s MVC, F-wave are was reduced by 35.9±18.9% for the placebo condition and 54.9±25.3% for the paroxetine condition. The occurrence of F-waves following the 60-s MVC significantly decreased by 31.8±15.6% for the placebo and 48.1±22.1% for paroxetine. F-wave persistence was also significantly decreased. |
| Kim et al. (44) | The clozapine group demonstrated higher resting heart rate compared with the non-clozapine (99.1±9.4 vs. 88.1±15.3 bpm, p=0.062) and higher peak heart rate (126.5±19.3 vs. 149.9 ± 24.2 bpm, p<0.01). The clozapine had lower heart rate reserve than the non-clozapine (27.5±16.8 vs. 61.7± 1.6, p<0.01). There was no significant difference in RPE between the clozapine group vs the non-clozapine group (8.5±1.8 vs. 8.3±1.7; p=0.864) as well as in respiratory exchange ratio (p=0.503). The clozapine group demonstrated significantly lower VO_2_peak than the non-clozapine group (F_1,27_ =4.47, p=0.044, η_p_^2^=0.14). No significant difference in oxygen pulse and ventilation was found. |
| King et al. (44) | Mean grip force over all trials was significantly higher in methylphenidate than placebo conditions (t=2.38, P=0.032, Cohen"s d=0.162, 120.8±38.17 vs 127.3±41.93 N). |
| Klass et al. (33) | Compared with placebo (35.79±11.74 min), reboxetine (30.21±6.96 min) reduced the endurance time by 15.6% (P=0.04). At task failure, MVC decreased by 41.1% in reboxetine and 43.6% placebo condition, whereas the rate of decline was greater in reboxetine than that in placebo condition (P=0.02). At the end of the fatigue test, RPE reached maximal value for all subjects in both conditions but the mean rate of change over time was significantly greater (P<0.01) in reboxetine condition. Heart rate increased during the fatigue test and reached 143.6±14.8 bpm in reboxetine and 127.2±14.1 bpm in placebo condition at task failure. |
| Klass et al. (32) | Subjects in reboxetine condition took 9.4% more time to complete the time trial in (33.66±3.61 min) compared with the placebo condition (30.78±2.08 min; P<0.05). Time needed to complete the time trial was not significantly different between methylphenidate (29.73±1.40 min) and placebo trials (P=0.19). Heart rate was higher for methylphenidate (185.4±7.3 bpm) than for placebo (174.4±9.2 bpm, P=0.05) at the end of the time trial, but there was no significant effect for reboxetine (168.8±16.6 bpm, P=0.40). RPE values at the end of the time trial was similar between placebo (18.0±1.4), reboxetine (18.1±1.1), and methylphenidate (18.7±1.1) conditions (P=0.18). The mean power output generated during the time trial was significantly (P<0.05) lower in the reboxetine trial compared with the placebo trial (252±31 vs 271±26 W), whereas no difference (P=0.18) was observed between methylphenidate (279±28 W) and placebo trials. The MVC torque was significantly reduced for the three conditions when recorded 10 min after the end of the time trial (post hoc tests, P<0.05). |
| Meeusen et al. (36) | Time to exhaustion was not different between the trials (p>0.05). Heart rate at exhaustion was similar in all trials (maximum increase 264% placebo and 268% Ritanserin). Respiratory quotient at the end of exercise was significantly higher in the Ritanserin group, while the increase during exercise was comparable in all groups (about 20% increase in all trials). Lactate increased during exercise in all trials and was comparable in all trials. |
| Meeusen et al. (25) | Exercise time did not differ between all three trials (p1acebo 1: 93.3±7.4 min, placebo 2: 87.9±7.1 min, fluoxetine: 88.8±10 min). There was no statistically significant difference in RPE rates between the trials (for whole body, placebo 1: 3.6±0.9, placebo 2: 4.2±1.5, fluoxetine: 3.9±1.3) and for the RPE rated over the legs (placebo 1: 3.2±0.9, placebo 2: 3.7±1.4, fluoxetine: 3.3+1.2). Maximal lactate concentrations were different from the resting values only in the fluoxetine trial (2.93±0.3 mmol/l vs. 0.7±0.7 mmol/l) while no statistical difference was observed in the placebo 2 trial. |
| Onus et al. (28) | Fixed intensity: There were no significant differences in total distance cycled, mean and peak power output, mean speed and cadence between bupropion and placebo conditions. Maximum speed during the sprints was significantly higher in placebo compared with bupropion (20°C: 64.9±3.0 vs. 66.3±1.0 km/h and 32°C: 62.8±5.4 vs. 66.4±1.4 km/h; P<0.05). In moderate environment (20°), maximum speed during sprints was approximately 1.4 km/h (P<0.05) faster under placebo than under bupropion. In warm environment (32°), maximum speed during sprints was approximately 3.2 km/h (P<0.05) faster under placebo than under bupropion.  Time trial (self-paced): There were no significant differences amongst conditions for total distance, mean power output, maximum speed during the low intensity efforts and mean cadence. The maximum speed during the sprints was similar between conditions. |
| Parise et al. (17) | Acute Study: MVC torque was higher in the placebo, compared with the fluoxetine condition (fluoxetine, 202.7±50.7 Nm; placebo, 231.6±30.3 Nm; p=.05). Absolute peak power (APP) and relative peak power (RPP) did not differ significantly between treatments (p>.05). In the placebo condition, there was a 4.2% and 4.3% decrease (p<.01), whereas in the fluoxetine trial there was only a 3.0% and 2.8% decrease (p>.05) in APP and RPP, respectively. There was no difference in the time to exhaustion (p>.05), mean power (p>.05), fatigue index and blood lactate between treatments. Chronic Study: MVC did not differ between trials (p>.05). Peak and mean power did not differ significantly between the fluoxetine and placebo trials (p>.05). Blood lactate concentration 2 min post exercise did not differ. VO_2_max did not differ significantly between conditions, nor was there a difference between conditions for fatigue (p>.05). Ventilation was lower during the 90% VO2max trial for fluoxetine compared with placebo (fluoxetine, 86.3±16.0L/min; placebo, 92.7±16.3L/ min; p>.05). There was no significant effect for treatment on peak or mean heart rate during the 90% VO_2_max trial. |
| Perez-Cruzado et al. (38) | Regarding risperidone, significant differences were found only in aerobic condition (2-minute step test) in risperidone consumer (111.03±16.94 bpm) compared to non-consumer (108.38±32.63 bpm) (p<0.05). Regarding olanzapine, significant differences were found for muscle strength, balance and aerobic condition in olanzapine consumers compared to non-consumer (p<0.05). Please refer to the original paper for the complete table. |
| Piacentini et al. (34) | Exercise time did not differ between trials (reboxetine: 97±3 min, placebo: 92±1 min; P=0.2). Lactate did not differ between trials. Heart rate during exercise did not differ between trials (max. heart rate: 177±9.5 bpm reboxetine vs 177±8.6 bpm placebo). RPE measured at the end of exercise was not different between trials (reboxetine: 8.1±0.9, placebo: 7.7±1). |
| Piacentini et al. (29) | Exercise time was not influenced by bupropion (placebo: 89±1 min; bupropion: 89±0.7 min). RPE values during exercise showed no differences between trials. Lactate concentrations and heart rate did not differ between trials. |
| Roelands et al. (35) | Subjects finished 10% and 20% slower with reboxetine in temperate and warm condition compared with the placebo (Pla18: 29 min 54 s ± 1 min 18 s; Rebox18: 32 min 54 s ± 3 min 42 s; Pla30: 40 min 36 s ± 6 min 24 s; Rebox30: 48 min 36 s ± 10 min 54 s). In both the Pla18 and Pla30 time trials, the power output was significantly higher than in the respective Rebox time trials (Pla18: 250.7±30.9 W; Rebox18: 230.5±39.7 W; P=0.009; Pla30: 189.6±49.2 W; Rebox30: 166.3±51.9 W; P=0.007). In Rebox 18 heart rate remained elevated above the values of the placebo trial. In the heat, no effect of drug treatment was reported. At the end of the time trial (*P=*0.025; Pla18: 6.32±2.62 mmol/l; Rebox18: 3.65±0.93 mmol/l) and after recovery (*P=*0.002; Pla18: 4.07±1.48 mmol/l; Rebox18: 2.49±0.84 mmol/l) in Rebox18, blood lactate concentrations were significantly lower than during Pla18. |
| Roelands et al. (18) | Subjects finished the placebo time trial after 41’36”±1’54” and the bupropion time trial after 40’42”±1’30” (P=0.659). Mean power output in the bupropion trial was 196±6 W, in the placebo trial 206 ± 4 W. Heart rates in both conditions increased over time (P=0.001), without any influence of the drug administration. RPE values were similar in both trials. |
| Roelands et al. (45) | Time trial was completed 16% faster in methylphenidate (38.1±6.4 min) than in placebo (45.4±7.3 min; P=0.049) in the heat (30°C). Throughout methylphenidate (30°), heart rates were significantly higher (P<0.05). In the 30°C trials, power output was 226±37 W in methylphenidate and 196±35 W in placebo until completion of the target amount of work (P=0.028). RPE values were similar between placebo and methylphenidate treatment in temperate and warm conditions, despite a higher RPE after 60 min in the warm placebo trial (P=0.100 in 18°C and P=0.386 in 30°C). |
| Roelands et al. (30) | Subjects finished the time trial faster in bupropion 300 mg compared with placebo (P=0.035; placebo: 33’42”±2’12”, bup100: 32’06”±1’54“).In bupropion 300 mg, heart rate showed a tendency to be higher near the end of exercise (placebo: 180±15 b/min; bup100: 186±7 b/min). RPE was similar between the placebo and different bupropion treatments. |
| Strachan et al. (20) | Time to exhaustion was not (P=0.66) influenced by administration of paroxetine. Subjects cycled for 93.3 (76.2–175.0) min on the placebo trial and 92.5 (66.0–151.0) min on the paroxetine trial. RPE and heart rate increased with exercise but were not different between trials. Blood lactate was not different between trials. |
| Strüder et al. (21) | Exhaustion was reached significantly earlier after paroxetine administration (131±36 min) compared to placebo trial (157±53 min). Heart rate and blood lactate did not show any difference to placebo trial. |
| Suda et al. (43) | There were no significant differences between eszopiclone and placebo conditions in vertical jump, 50-m sprint and repeated side jumps (p=0.047). Vertical jumps was 53.8±8.3 cm in eszopiclone and 54.8±8.4 cm in placebo. 50-m-sprint was 7.6±0.6 s in both. Side jumps was 46.6±5.3 n in eszopiclone and 47.3±5.6 in placebo. |
| Tafti et al. | There were no significant differences between zopiclone and placebo in standing jump test (325.86±1.53 vs. 325.52±1.48) and in running time (277.09±1.87 vs. 279.90±1.87 ms). |
| Teixeira-Coelho et al. (22) | Two groups with higher (HAC) and lower (LAC) aerobic capacities: None of the paroxetine doses influenced the total exercise time in the LAC group. For the HAC group, the total exercise time in the 20 mg paroxetine condition was 15% less than that in the placebo condition (76.3±5.1 min vs. 90.0±7.9 min; p<0.05). No differences were observed in heart rate among the experimental trials or between the groups. Administration of 40 mg of paroxetine induced exaggerated increases in the RPE of subjects from the LAC group when compared to subjects in the HAC group. |
| Thorstensen et al. (23) | Paroxetine did not influence torque or voluntary activation during brief MVCs performed throughout the low-intensity contraction. Paroxetine increased the perception of fatigue throughout the contraction (P=0.005) and shortened the biceps silent period elicited (P=0.003) and brief MVCs (P=0.011). |
| Watson et al. (4) | In the temperate condition (18°C), no differences in exercise time were found (placebo: 30.6±2.2 min vs. bupropion: 30.6±1.9 min) (P=0.954). Subjects completed the time trial 9% faster in the bupropion (36.4±5.7 min) than in the placebo trial (39.8±3.9 min; P=0.046) in the warm environment (30°C). Mean power output was greater throughout the bupropion trial in the warm condition (234±35 W) than during the placebo trial (211±23 W). At the end of the time trial in the heat, heart rate (pla 178±7 bpm, bup 183±12 bpm; P=0.039) was higher in the bupropion trial than in the placebo. |
| Westover et al. (46) | The pattern of the adjusted least squares means showed that peak heart rate was significantly lower for users than for nonusers [170.2 (SE=2.02) vs. 174.4 (SE=1.92), raw p<0.0001, FDR-adjusted p=0.0004]. Stimulant medication use was not associated with changes in peak SBP, average SBP rise, or estimated VO_2_max. |
| Wilson et al. (24) | Exercise time after paroxetine (median 94 min; range 84-127 min) was less (P<0.05) than after placebo (median 116 min; range 86-133 min). Heart rate was the same at all time points in both trials. Blood lactate peak was 3.1±1.2 mml/l in placebo and 3.4±1.6 mml/l in paroxetine. RPE was not different between trials. |
